# Ethnobotanical survey of plants used in Afyonkarahisar-Turkey

**DOI:** 10.1186/s13002-015-0067-6

**Published:** 2015-12-23

**Authors:** Süleyman Arı, Mehmet Temel, Mustafa Kargıoğlu, Muhsin Konuk

**Affiliations:** Department of Molecular Biology and Genetics, Science & Arts Faculty, Afyon Kocatepe University, 03200 Afyonkarahisar, Turkey; Department of Molecular Biology and Genetics, Faculty of Engineering and Natural Sciences, Üsküdar University, 34662 Istanbul, Turkey

**Keywords:** Afyonkarahisar, Ethnobotany, Food plants, Medicinal plants

## Abstract

**Background:**

The traditional knowledge about plants and their uses in Turkey is disappearing in recent years because the new generations of villagers migrate to big cities for a better life. Afyonkarahisar located at the intersection of roads and phytogeographical regions (Mediterranean, Iran-Turan, and Euro-Siberian) has more than 2500 plant species. This richness of plant diversity promotes the indigenous commuity for the traditional use of wild plants. The aim of the study is to show wild plants’ ethnobotanical usages associated with medicinal, food, fodder, and household goods in 31 settlements within the boundaries of Afyonkarahisar province.

**Methods:**

The ethnobotanical data were collected from 46 informants by means of semi-structured interviews from 2012 to 2014. Ethnobotanical uses of plants of the study area were conducted in the vicinity of Afyonkarahisar (5 districts, 8 towns, 15 villages, and 3 neighborhood centers).

**Results:**

One hundred and thirty plant taxa belonging to 39 families were recorded and collected. Hundred and seventy-eight different uses of these plants were documented and used generally for medicinal (84), food (68), fodder (16), household goods (3), dyes (3), handicrafts (3) and religious (1).

**Conclusion:**

This study provides interesting uses of plants in the local community of Afyonkarahisar and its surrounding area, in what purpose they make use of plants, how they make use of them and obtained results will contribute to economy of villagers. Since the local people, especially in villages, are poor and do not have health care, they use the plants to treat illnesses, food, fodder, household goods and other uses (evil eye). Also this study will light the way for posterity for next generations.

## Background

People have interacted with plants since ancient times. This interaction has contributed to flourishing of scientific fields such as ethnobotany and paleoethnobotany [1]. Ethnobotanical studies began in the early 1800s when John W. Harsberger, a famous botanist, proposed ethnobotanical study for the first time [2]. The scope of plant use has changed since the 1800s to this day. The frequency and purpose of use of plants by people vary in regard to social, cultural, and economic needs. Plants are used for purposes of food, medicine, fuel, industry, ornament, and effects. Purposes of use also vary in regard to people’s priority of needs [1, 3–9]. Turkey, with more than 11,000 taxa is a flora-rich country due to its climate and phytogeographical positions (Mediterranean, Iran-Turan, and Euro-Siberian) is a significant position as being a flora-rich country. The endemic plants in its flora occupy 1/3 of total taxa. Anatolian people have been using these plants as food and medicine since Paleolithic times [10, 11]. Approximately 1000 taxa are used for medicinal purposes and 350 plant species are used in internal and external trade [12]. Afyonkarahisar is located where the three regions intersect. This makes Afyonkarahisar a flora rich region, people use the plants arund their environment for different purposes.

Turkish people living in rural areas use especially wild plants. Generally, the usage of plants are for food and medical purposes. In recent years, traditional ethnobotanical knowledge and prevalence of medicinal plants have been investigated by researchers in different areas of Turkey [13–41]. As a results of these studies a great increase on the level of traditional knowledge of plants occured. On the other hand, more detailed studies are needed to focus region by region. Therefore this study was carried out to extend Afyonkarahisar’s ethnobotanical knowledge due to a limited ethnobotanical studies [42–46] conducted in the near region; living in suburbs and in villages; protecting and maintaining their traditional culture and customs and rich uses of plants by local people. The aims of this study were: (1) to determine the local and scientific names of the plants, (2) to document and analyse the traditional ethnobotanical knowledge herited by local people living in Afyonkarahisar and its surrounding area.

## Methods

### Study Area

**Fig. 1:**
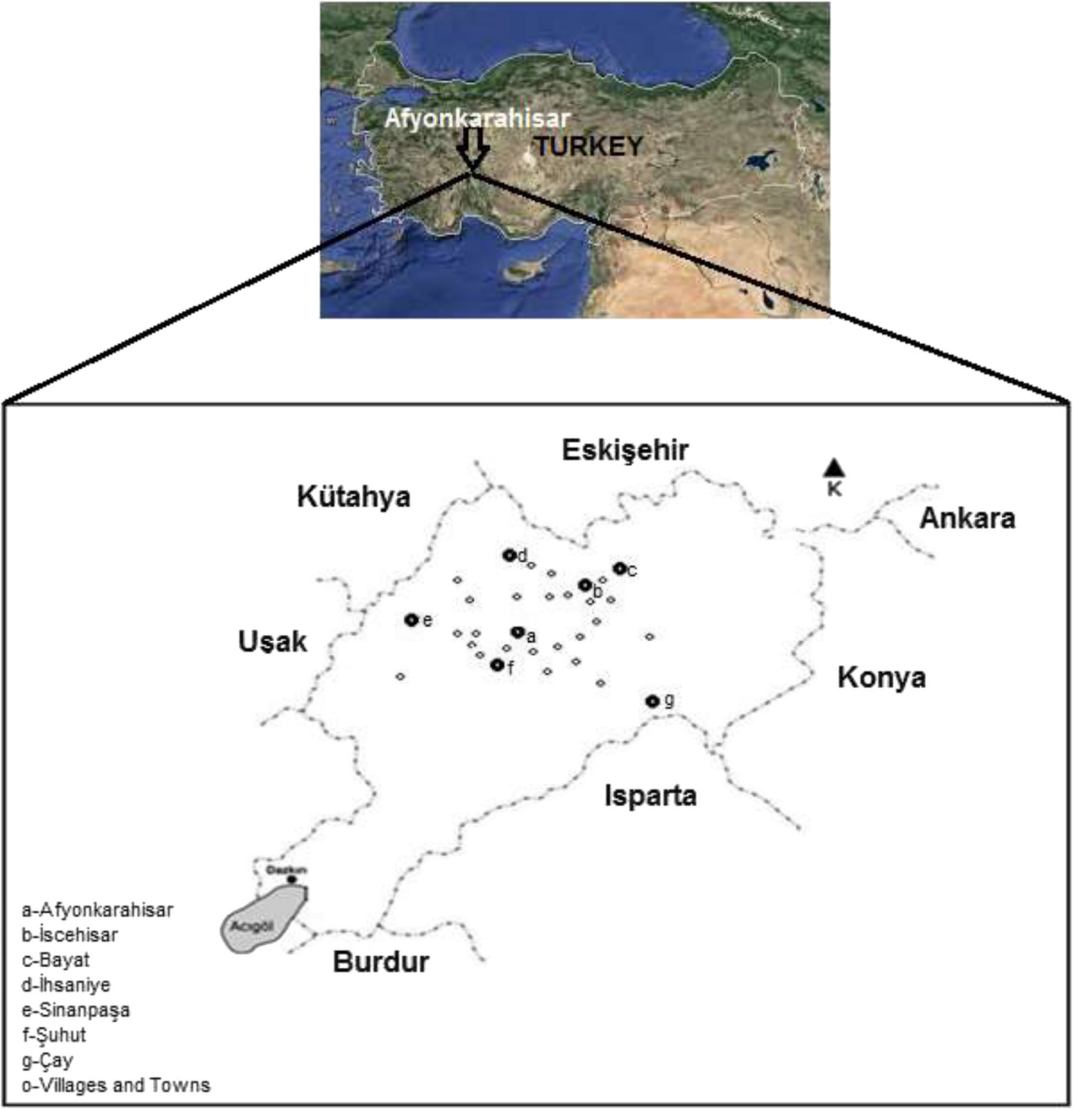
The study area and Afyonkarahisar’s location map

Afyonkarahisar is 1034 m above sea level. It is located 38° 45‘N latitude and 30° 32’ E longitude. The total area of Afyonkarahisar is 14,295 km^2^ and it occupies 1.8 % of Turkey’s land. In north of Eskişehir, northwest of Kütahya, east of Konya, south of Isparta, west of Uşak, southwest of Denizli and Burdur are located (Fig. 1) [46]. Despite the fact that Afyonkarahisar is located in the Aegean region, its climate is similar to that of the central Anatolia region. Winters are cold and tough with intense snow, summers are hot and dry, and spring and autumn months feature rain. Precipitation is raining in spring and autumn [47]. According to Erinç [48], the index value of Afyonkarahisar is 23.9 lm. In the vegetation of Afyonkarahisar, cedar and blackpine are found along with various species including relict ones. However, blackpine forests, the dominant factor of forest formation, have been significantly destroyed and oak groups have replaced them. The destruction is greater especially in fields around settlements, and these fields have turned into anthropogenic steppe [47]. The main livelihoods of the local community in the research area are tree felling, sheep and cattle husbandry, and agriculture. Animal husbandry consists of small numbers of cattle per household (average one), kept for meat and milk, with dairy products being sold in local bazaars. Since the area consists largely of forested hillsides, crop production is restricted to small fields, and annual incomes from agriculture are therefore relatively low. Monthly incomes are in the region of US $230–350 for workers and shepherds, and $350 for agricultural workers in those months that they work. On average, 50 % of the population is young (under 30 years), 30 % are middle-aged (30–50 years), and 20 % are old (50+ years). Although 80 % of the middle-aged and nearly 80 % of the older generation is not literate, almost all young people are literate.

### Data collection

Specimens were collected by the authors in Afyonkarahisar and its surrounding area in the years between 2012 and 2014. Thirty-one settlements were visited for field research. Two hundred people were contacted, and 46 of them accepted to become our informants who have ethnobotanical experience. Thirty-five of them were male and 11 of them were female. Data were collected from nine informants between the ages of 35 and 50, 17 informants between the ages of 50–65, and 20 informants over the age of 65. Interviews with the men were usually carried out in the teahouses where they come together, and with women in their homes, bazaars and gardens. A questionnaire was administered to the informants through face-to-face interviews. Information that had been carried to the region from the outside and that was not used or confirmed were not included and recorded. During the interviews, the below questions were asked to the participants.

1. Name and surname
2. Age and sex
3. Educational level
4. Are plants collected in your region?
5. Do you have any contact with plants?
6. Can you show the plants you use in your region?
7. Can you tell the local names of the plants you use in your region?
8. In which season do you collect the plants you use in your region?
9. When collecting plant, which parts of the plant do you collect and how do you collect them?
10. Which parts of the plants do you use? (Flower, fruit, leaves, root, tuber, young shoots, branch, galbula, cupula, stem, above ground parts etc.).
11. How do you prepare and administrate the plants’ parts?

Answers given above questions with doubt were not recorded. Specimens were collected and identified by the authors according to Davis [49] and the studies related to the flora Afyonkarahisar by Kargıoğlu et al. [44, 45]. Plants were photographed as well as being observed in the research field. Voucher specimens are saved in the Herbarium of Afyon Kocatepe University (AKUH). Herbarium numbers of the plant taxa were given in Table 1.

**Table 1:** List of plants used as foodstuff or medicinal purposes in Afyonkarahisar (Inner-West Anatolia)

Species	Plant Family	Local Name	Parts Used	Uses	Preparation and Administration
*Acanthus hirsutus* Boiss. (AKUH 7506)	Acanthaceae	Ayıpençesi	Stem, Leaf	Fodder	Stem and leaf are consumed by animals for fodder.
*Amaranthus retroflexus* L. (AKUH 7509)	Amaranthaceae	Paşa pancarı, kızılbacak	Leaf	Food	The plant’s leaves are fried in oil and consumed.
*Conium maculatum* L. (AKUH 7520)	Apiaceae	Yılan kamışı, gumarcık ot	Flower	Infection	The plant oil were removed and the stem is driven to kill germs in the stem structure.
*Eryngium campestre* L. var. *virens* Link. (AKUH 7528)		Çakırdikeni	Stem	Infection	Infusion as tea.
*Arum elongatum* Steven subsp. *elongatum* Steven (AKUH 7542)	Araceae	Basur otu	Root, Tuber	Hemorrhoid	Plant tuber part turned into powder by in effect simulating the board. The capsule was consumed.
*Dracunculus vulgaris* Schoot. (AKUH 7564)		Yılan bıçağı	Leaf	Infection	The leaf part is used as a salve on a wound.
*Muscari comosum* (L.) Mill. (AKUH 7597)	Asparagaceae	Dağ soğanı, ada soğanı	Above ground parts	Circulatory system	Infusion as tea.
*Tragopogon latifolius* Boiss. var. *angustifolius* Boiss. (AKUH 7514)	Asteraceae	Tekesakalı, yemlik	Leaf	Stomach disease	The leaves are directly consumed.
*Helianthus tuberosus* L. (AKUH 7521)		Yerelması	Root, Stem	Food	Root and stem parts are directly consumed.
*Anthemis tinctoria* L. var. *tinctoria* L. (AKUH 7545)		Papatya	Flower	Respiratory system	Infusion as tea.
*Hieracium pannosum* Boiss. (AKUH 7548)		Sakız otu	Root	Oral and dental health	The root of the plant is suspended in the sun, the resulting liquid such as milk dry up like chewing gum is for chewing.
*Anthemis wallii* Hub.-Mor. et Reese (AKUH 7501)		Papatya	Flower	Asthma and shortness of breath	Infusion as tea.
*Chondrilla juncea* L. var. *juncea.* (AKUH 7504)		Karakavuk, çıtlık, çengel sakızı	Leaf	Painkiller and stomach disease	The leaves are used in salad.
*Lactuca serriola* L. (AKUH 7502)		Tarla marul, acı marul, dilli tura	Leaf	Diet and attenuator	The leaves of the plant are consumed as part of a salad.
*Achillea millefolium* L. subsp. *millefolium.* (AKUH 7534)		Ayva denesi	Leaf, Flower	Diseases of the digestive system	Infusion as tea.
*Achillea teretifolia* Willd. (AKUH 7532)		Yaraotu	Above ground parts	Diseases of the digestive, skin and acne	The plant’s above ground parts boiled water by putting a quantity of 15 min is suspended. Tea mixed with oil is applied on acne and wounded regions.
*Centaurea depressa* M. Bieb. (AKUH 7560)		Gökbaş	Leaf	Food	The leaves of the plant are consumed by making a taco.
*Cichorium intybus* L. (AKUH 7557)		Acı günek, çiftlik otu	Leaf	Painkiller and stomach diseases	The plant’s leaves are made of salad and rolls.
*Scolymus hispanicus* L. (AKUH 7503)		Diken	Stem	Digestive diseases	The fleshy parts of the stem of the plant is consumed directly in the blister pack
*Bellis perennis* L. (AKUH 7511)		Çayır papatyası	Flower	Medical, respiratory diseases	Flower of the plant is dried, boiled water for 3–5 min by joining strength, juice drink.
*Doronicum orientale* Hoffm. (AKUH 7513)		Sarıçiçek	Flower, Leaf	Fodder	The plant flowers and leaves parts exposed to animals as bait.
*Onopordum anatolicum* (Boiss.) Eig. (AKUH 7562)		Galgan	Stem	Digestive tract, stomachache, kidney stones	The meaty parts in the stem of the plant is removed, directly from the defeated are consumed. Decoction as tea.
*Gundelia tournefortii* L. var. *tournefortii* (AKUH 7556)		Kenger	Fruit	Skin disorders, eczema, hemorrhoids	After roasting, the fruit portion is consumed.
*Artemisia campestris* L. (AKUH 7589)		Pelin	Young shoots and Leaves	Appetizer	Decoction as tea.
*Achillea biebersteinii* Afan. (AKUH 7573)		Civanperçem	Leaf, Flower	Pain relievers, stomach, respiratory distress, shortness of breath disorders	Decoction as tea.
*Inula anatolica* Boiss.(AKUH 7576)		Basur otu	Flower	Hemorrhoids	Flower is boiled in water. It is used as a treatment for hemorrhoids district.
*Berberis crataegina* DC.(AKUH 7629)	Berberidaceae	Sarıçalı	Leaf, Fruit	Kidney stones, liver	Leaves are effective for preventing kidney stones when they are consumed 15–20 days as salad. The fruit of the plant part (grape) is consumed directly.
*Anchusa leptophylla* Roemer & Schultes subsp*. leptophylla* (AKUH 7505)	Boraginaceae	Ballık Otu, Kuzu dili	Stem, Flower,	Food	Stem and flowers are put into the dough.
*Cerinthe minor* L. subsp. *auriculata* (Ten.) Domac (AKUH 7558)		Sarıçiçek	Flower, Leaf	Fodder	Leaves and flower parts are exposed to animals.
*Anchusa undulata* L. subsp. *hybrida* (Ten.) Coutinho (AKUH 7552)		Sığırdili	Leaf	Diabetes	The plant’s leaves are boiled and are consumed by joining into the dough.
*Alkanna tinctoria* (L.) Tausch subsp*. glandulosa* Hub.-Mor. (AKUH 7561)		Havacıva otu	Root	Hemorrhoids	Decoction as tea, the region also has a therapeutic equivalent of hemorrhoids.
*Anchusa azurea* Mill. var. *azurea.* (AKUH 7553)		Kuzu dili, Ballık otu	Leaf	Food	Fresh leaves of the plant are boiled and put into dough.
*Alyssum desertorum* Stapf. var. *desertorum* Stapf. (AKUH 7554)	Brassicaceae	Yozmercimek	Fruit	Food	Consumed directly by shepherds.
*Sisymbrium altissimum* L. (AKUH 7590)		Hardal otu	Leaf, Flower	Food, Fodder	Leaf of the plant parts consumed in the form of rolls wrapped in phyllo dough. Leaves and flowers of the plant are given to animals.
*Barbarea* sp. (AKUH 7583)		Acı tere	Leaf	Body resistance, vitamin	The plant’s leaves are made of salad.
*Capsella bursa-pastoris* (L.) Medik. (AKUH 7507)		Pastariz, bicibici	Leaf	Food	The plant fresh leaves are consumed in the form of pastry wrapped into rolls.
*Sinapis arvensis* L. (AKUH 7508)		Hardal	Leaf	Food	Boiled fresh leaves is consumed in taco.
*Silene dichotoma* Ehrh*.* subsp. *dichotoma* Ehrh. (AKUH 7512)	Caryophyllaceae	Toklubaşı	Leaf	Food	The plant’s leaves are made of salad
*Stellaria media* (L.) Vill*.* subsp*. media.* (AKUH 7510)		Kuşekmeği, urgancık, kazayağı	Leaf	Food	The leaves of the plant is consumed wrapped in dough.
*Silene vulgaris* (Moench) Garcke var. *vulgaris* (AKUH 7515)		Toklubaşı	Leaf	Food	Fried in oil and consumed.
*Agrostemma githago* L. (AKUH 7628)		Sakızlık otu	Flower	Digestive disorder	Infusion as tea.
*Dianthus zonatus* Fenzl. var*. aristatus* (Boiss.) Reeve (AKUH 7620)		Basur otu	Flower	Hemorrhoids	Flower part is boiled in water in 3–5 min. It is drunk for hemorrhoidal disease by the use of 10–15 sessions
*Dianthus zonatus* Fenzl var*. zonatus* (AKUH 7598)		Siğilotu	Flower	Skin diseases, warts	Infusion as tea.
*Vaccaria pyramidata* Medik. var. *grandiflora* (Fisch. ex DC.) Cullen (AKUH 7623)		Mor çiçek	Flower, Leaf	Fodder	It is consumed as fresh by animals..
*Chenopodium album* L. (AKUH 7588)	Chenopodiaceae	Sirken	Leaf	Food	The plant’s leaves are boiled, consumed in dough.
*Chenopodium album* L. subsp. *album* var. *album* (AKUH 7582)		Sarı sirken	Leaf	Food	The plant’s leaves are roasted and the eggs are added on to it.
*Chenopodium foliosum* (Moench) Asch*.* (AKUH 7630)		İt üzümü	Fruit	Food	Fruits are eaten in fresh.
*Beta trigyna* Waldst. & Kit. (AKUH 7625)		Kır ıspanağı	Leaf	Digestive and stomach diseases	Fried in oil and eaten by shepherds.
*Kochia scoparia* (L.) Schrad. (AKUH 7626)		Süpürge	Branch, Stem	Household goods	Turned into a broom is used as household goods.
*Atriplex* sp. (AKUH 7627)		Tellice	Flower	Immune system	Infusion as tea.
*Cistus laurifolius* L. (AKUH 7624)	Cistaceae	Pinar	Leaf, Young shoots	Crafts, coloring, digestive	The leaves and young branches of the plant are boiled, green, yellow and tones are obtained. The leaves and shoots are boiled in water to drink.
*Juniperus oxycedrus* L. subsp*. oxycedrus* (AKUH 7622)	Cupressaceae	Gıli gıli	Leaf, Galbula	Cholesterol, diabetes	The plant’s fruit and leaves are boiled in water for 10–15 min for a drink. Fresh fruits are edible or boiled to prepare juice.
*Juniperus excelsa* M. Bieb. (AKUH 7621)		Katran ağacı	Stem	Digestive and infection diseases	The plant body part turned into tar at high temperature and used for cleaning of the infection and digestive problems.
*Juniperus foetidissima* Willd. (AKUH 7619)		Kokar ardıcı	Leaf	Skin diseases, warts	*Juniper* leaves, broken in and they are applied into the warty zone 30–40 sessions.
*Equisetum ramosissimum* Desf. (AKUH 7617)	Equisetaceae	Kırk kilit	Stem	Respiratory, sinusitis and arthritis diseases	Decoction as tea.
*Euphorbia macroclada* (Boiss.) (AKUH 7616)	Euphorbiaceae	Sütleğen	Stem	Infection	The plant is removed from the body in the form of liquid milk. Liquid bread into the stained area and ingested for treatment malaria.
*Vicia cracca* L. subsp. *stenophylla* Velen. (AKUH 7613)	Fabaceae	Efek	Flower, Fruit	Food, fodder	Flowers and fruits are consumed directly.
*Astragalus flavescens* Boiss. (AKUH 7618)		Eşek geveni	Leaf, Flower	Fodder	The plant’s leaves and flower parts are consumed directly by animals
*Astragalus microcephalus* Willd. (AKUH 7615)		Geven	Above ground parts	Fodder	Spiny part is burned by shepherds. A hammer or mallet were crushed for animals to eat.
*Coronilla varia* L. subsp. *varia* (AKUH 7611)		Burçak	Flower, Leaf	Respiratory diseases	Infusion as tea.
*Astragalus pisidicus* Boiss. & Heldr. (AKUH 7612)		Söğüt geveni	Above ground parts	Body resistance, immune system, cancer	Infusion as tea.
*Quercus ithaburensis* Decne. subsp. *macrolepis* (Kotsch) Hedge&Yalt. (AKUH 7614)	Fagaceae	Palamut	Cupula of the plant	Crafts, painting	Cupula of the plant by boiling chickpea yolk color is obtained for rug weaving.
*Quercus cerris* L. var*. cerris* (AKUH 7608)		Kızılmeşe	Fruit	Infection, hemorrhoids, Skin disorders, eczema	It is boiled in water and two spoons of juice is consumed on an empty stomach.
*Quercus infectoria* Oliv*.* subsp. *boissieri* (Reuter) O.Schwarz (AKUH 7610)		Gerçelik	Fruit	Fodder	Plant crops are exposed as sheep bait.
*Quercus pubescens* Willd. (AKUH 7595)		Tüylü meşe	Leaf	Fodder	The leaves of the plant are eaten by animals.
*Hypericum perforatum* L*.* (AKUH 7566)	Hypericaceae	Binbir otu	Above ground parts	Painkillers	Decoction as tea.
*Hypericum perfoliatum* L. (AKUH 7555)		Binbirdelik otu	Leaf, Flower	Digestive system	Decoction as tea.
*Juglans regia* L. (AKUH 7607)	Juglandaceae	Ceviz kabuğu	Fruit Peel	Skin cancer, crafts and coloring	Decoction as tea. It is boiled to obtain dark brown collors and tones for rug weaving.
*Tymus longicaulis* C. Presl subsp. *longicaulis* var. *subisophyllus* (Borbas) Jalas (AKUH 7601)	Lamiaceae	Dağ kekiği	Flower	Lowering cholesterol and sugar.	The plant is consumed in the form of oregano oil. Infusion as tea.
*Mentha longifolia* (L.) Huds*.* subsp. *typhoides* (Briq.) Harley var. *typhoides* (AKUH 7600)		Yabani nane, Doğuma	Leaf	Body resistance, vitamin, respiratory diseases	The plant’s leaves are consumed in salad Infusion as tea. Dried leaves of the plant are used for spices. Plant leaves are mixed to the dough.
*Teucrium chamaedrys* L. subsp. *chamaedry*. (AKUH 7603)		Bodurmamut, sancıotu	Leaf, Flower	Painkillers, stomach and hemorrhoid disease	Infusion as tea.
*Origanum vulgare* L. subsp. *hirtum* (Link) Ietsw*.* (AKUH 7599)		Dağ çayı	Flower	Digestive and stomach diseases	Infusion as tea.
*Thymus zygioides* Griseb. var*. lycaonicus* (AKUH 7606)		Mor kekik	Flower	Heart and vascular diseases	Infusion as tea.
*Salvia cryptantha* Montbret & Aucher ex Bentham (AKUH 7602)		Kır çayı, şapla	Leaf, Flower	Respiratory and colds	Infusion as tea.
*Marrubium globosum* Montbret et Aucher ex Bentham (AKUH 7578)		Oğul otu	Leaf	Cardiac, vascular diseases	Infusion as tea.
*Salvia tomentosa* Mill. (AKUH 7579)		Karakekik	Leaf, Flower	Food	Decoction as tea.
*Salvia virgata* Jacq. (AKUH 7592)		Kır kekiği	Flower	Food	Dried flowers are used for spices by joining tarhana soup
*Phlomis armeniaca* Willd. (AKUH 7593)		Zorlatma otu	Flower	Painkillers	Flower oil is applied to the pain region.
*Thymus sipyleus* Boiss. subsp*. sipyleus* var. *sipyleus* (AKUH 7604)		Beyaz kekik	Flower	Respiratory diseases shortness of breath, influenza	Infusion as tea.
*Mentha pulegium* L. (AKUH 7605)		Yarpuz	Flower	Food	Dried flowers of the herb is consumed as spices. Infusion as tea.
*Teucrium polium* L. (AKUH 7591)		Acı ot	Stem	Hemorrhoids	Infusion as tea.
*Linum hirsutum* L. subsp. *anatolicum* (Boiss.) Hayek var*. anatolicum.*(AKUH 7596)	Linaceae	Keten	Flower	Food	The flowers of the plant are consumed directly.
*Viscum album* L. subsp*. album* (AKUH 7594)	Santalaceae	Bögem, burç	Leaf, Young shoots	Respiratory, cough, digestive, intestinal gas reliever	Infusion as tea.
*Arceuthobium oxycedri* (Dc.) M. Bieb. (AKUH 7584)		Ardıç burçu	Stem	Neurological diseases	Decoction as tea.
*Malva sylvestris* L. (AKUH 7587)	Malvaceae	Ebegümeci	Leaf	Food	After rosting in oil, it is consumed in the form of food.
*Malva neglecta* Wallr. (AKUH 7586)		Ebegümeci	Leaf	Painkiller	Infusion as tea. Fresh leaves of the plant participates in the dough.
*Morus nigra* L*.*(AKUH 7585)	Moraceae	Doğal dut	Fruit	Infection, aphthae	Marmalade is made from fruit.
*Peganum harmala* L. (AKUH 7572)	Nitrariceae	Üzerlik	Above ground parts	The evil eye	It is believed to prevent for the evil eye to strike the bride and son-in-law.
*Chelidonium majus* L. (AKUH 7563)		Kırlangıç otu	Above ground parts	Digestion, hemorrhoids, liver, jaundice, eye diseases, skin diseases	Infusion as tea (1–2 cups a day)
*Fumaria asepala* Boiss*.* (AKUH 7543)		Şahtere	Flower	Iinfection, fungus	Infusion as tea. The plant’s water is applied to fungal region.
*Papaver dubium* L. (AKUH 7551)		Yaban haşhaşı	Leaf	Food	The leaves of the plant are consumed by making a salad.
*Pinus nigra* Arn. subsp. *pallasiana* (Lamb.) Holmboe var*. pallasiana* (AKUH 7565)	Pinaceae	Katran çamı	Stem	Infection	Tar in water is drunk for infection.
*Plantago lanceolata* L. (AKUH 7525)	Plantaginaceae	Sinirli yaprak	Leaf	Infection	Leaf of the plant part is driven directly to the inflamed area. It is used for cleaning of the infection.
*Plantago major* L. subsp. *intermedia* (Gilib.) Lange (AKUH 7531)		Yılanotu	Leaf	Infection	Leaf of the plant part affected area to be wrapped, provides to outside infection.
*Acantholimon ulicinum* (Willd. & Schultes) Boiss*.* subsp*. lycaonicum* (Boiss. & Heldr.) Bokhari. & Edm. (AKUH 7580)	Plumbaginaceae	Porsuk	Flower, Leaf	Household goods	Used in homes as decorative items.
*Acantholimon acerosum* subsp. *lycaonicum* (Willd.) Boiss. var. *acerosum* (AKUH 7581)		Keven, Porsuk	Flower	Household goods, infection, tuberculosis	Infusion as tea (1–2 cups a day)
*Rumex scutatus* L. (AKUH 7569)	Polygonaceae	Ekşimen	Leaf	Vitamin needs	The leaves of the plant are consumed directly. Phyllo dough is made between the rolls. Salad is made. It is consumed directly with salt.
*Rumex patientia* L. (AKUH 7571)		İlibada, Sabla	Leaf	Food	The leaf part is consumed as wheat wrapped (sarma). Leaf of the plant part participates in the dough.
*Rumex acetosella* L. (AKUH 7574)		Kuzukulağı	Leaf	Food	Leaves are eaten directly. Rolls are made, It is eaten.
*Rumex crispus* L. (AKUH 7575)		Evelik	Leaf	Food	The leaf part is consumed as wheat wrapped (sarma).
*Polygonum cognatum* Meissn. (AKUH 7518)		Çobanekmeği	Leaf	Body resistance	It is eaten as salad. Leaf of the plant part eaten directly.
*Rumex tuberosus* L.subsp. *tuberosus* L. (AKUH 7519)		Ekşikulak	Leaf	Body resistance, vitamin	It is eaten as salad. Leaf of the plant part eaten directly.
*Portulaca oleraceae* L. (AKUH 7522)	Portulacaceae	Temizlik otu	Above ground parts	Food	The plant’s above ground parts especially the leaves part joins into the yogurt. It is eaten as salad.
*Lysimachia vulgaris* L. (AKUH 7577)	Primulaceae	Mersin	Leaf	Digestive diseases	Infusion as tea (1–2 cups a day)
*Nigella sativa* L. (AKUH 7559)	Ranunculaceae	Çörek otu	Seed	Respiratory distress, shortness of breath, the immune diseases	The plant’s seed is consumed directly. Also, it is mixed into the honey.
*Ranunculus ficaria* L. subsp. *ficariiformis* Rouy & F. (AKUH 7567)		Sarıçiçek, Düğün çiçeği, Mayıs çiçeği	Flower	Digestion, hemorrhoids, skin diseases	Infusion as tea (3 cups a day)
*Adonis aestivalis* L. subsp. *aestivalis* L*.* (AKUH 7516)		Tavukgötü	Stem	Fodder	The stem of the plant parts are consumed by animals.
*Rhamnus rhodopeus* Velen. subsp*. anatolicus* (Grub.) Browicz & Zieliński (AKUH 7570)	Rhamnaceae	Yağlıcan çehri, Karaköken	Fruit	Debilitating, diabetes	Fruits of the plant part eaten directly.
*Pyrus elaeagnifolia* Pallas subsp*. elaeagnifolia* Pallas (AKUH 7568)	Rosaceae	Ahlât	Fruit	Cardiovascular diseases, hypertension	Infusion as tea(4 cups a day) Fruits of the plant part are eaten directly. Designated as a beverage.
*Rosa hemisphaerica* Herrm. (AKUH 7549)		Gündöndü çiçeği	Fruit	Food	Fruits of the plant part eaten directly.
*Rosa canina* L. (AKUH 7550)		Kuşburnu	Fruit	Urea treatment, hemorrhoids, gastric ulcer	Dried fruit is boiled for a long time in the water to get marmelata. The fruit is boiled and it is taken orally as cold drink. Infusion as tea(3–4 cups a day)
*Crataegus monogyna* Jacq. subsp. *monogyna.*(AKUH 7547)		Öküzgötü	Fruit	Respiratory, cold	Infusion as tea(2–4 cups a day) Dried fruit is boiled for a long time in the water to get marmelata.
*Prunus divaricata* Ledeb. subsp. *divaricata.*(AKUH 7541)		Yabani erik	Fruit	Body resistance	Fruits of the plant part are eaten directly.
*Geum urbanum* L. (AKUH 7535)		Dağçayı, meryemotu	Root	Respiratory, influenza	Decoction as tea.
*Crataegus aronia* (L.) Bosc*.* ex DC. (AKUH 7546)		Alıç	Fruit	Food	Fruits of the plant part are eaten directly.
*Malus sylvestris* Mill. subsp*. orientalis*(Uglitzk.) Browicz var. *orientalis* (AKUH 7540)		Dağ elması	Fruit	Food	The fruit of the plant is dried, boiled and drunk as juice.
*Crataegus orientalis* Pallas ex M. Bieb. var. *orientalis* (AKUH 7544)		Ahlât	Fruit, Young shoots	Diabetes, rheumatism	The ends of the branches and shoots of the plant parts are welded, cold drink. Fruits of the plant part are eaten directly.
*Cerasus vulgaris* Mill. (AKUH 7539)		Yozvişne	Fruit	Kidney, diuretic	Infusion as tea(8–10 cups a day) Compote is done from fruits.
*Prunus armeniaca* L. (AKUH 7533)		Kayısı kurusu	Fruit	Digestive and intestinal problems	Fruits of the plant part are eaten directly. Compote is done from fruits.
*Cotoneaster nummularia* Fisch. & C.A.Mey. (AKUH 7536)		Muşmula	Fruit	Food	Fresh fruits are boiled to prepare jam. Decoction as tea.
*Salix alba* L. (AKUH 7538)	Salicaceae	Söğüt	Leaf	Painkiller, stomach and respiratory diseases, shortness of breath	Infusion as tea(2 cups a day)
*Linaria genistifolia* (L.) Mill. subsp. *genistifolia* (AKUH 7537)	Scrophulariaceae	Geyşenik, Meryem otu	Flower	Fodder	Animals consume directly.
*Linaria genistifolia* (L.) Mill. subsp. *confertiflora* (AKUH 7517)		Geyşenik, Meryem otu	Leaf, Flower	Skin disorders, eczema	Portions of the leaves and flowers are boiled. It is applied 2–3 times a day for eczema areas.
*Verbascum* sp. (AKUH 7527)		Öküz kuyruğu, sığırkuyruğu	Leaf, Flower	Respiratory, asthma, shortness of breath, skin diseases, warts, eczema	Infusion as tea(2–4 cups a day) It is applied 2–3 times a day for eczema areas.
*Linaria grandiflora* Desf. (AKUH 7529)		Sarışın	Leaf, Flower	Fodder	Animals consume directly.
*Urtica dioica* L. (AKUH 7526)	Urticaceae	Isırgan	Leaf	Cancer, leukemia	Decoction as tea.
*Urtica urens* L. (AKUH 7530)		Dağlayan	Leaf	Skin cancer	Decoction as tea.
*Urtica pilulifera* L. (AKUH 7524)		Isırgan otu	Leaf	Food	Decoction as tea.
*Tribulus terrestris* L. (AKUH 7523)	Zygophyllaceae	Çoban çökerten	Flower, Leaf, Root	Kidney sand, hemorrhoids	Leaves are consumed to make taco. The flower oil is applied for hemorrhoids.

## Results and discussion

**Fig. 2:**
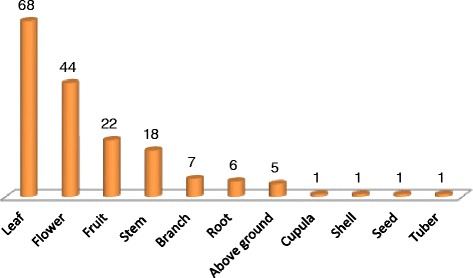
Used parts of the medicinal plants and their usage frequencies in the study area

As seen in Table 1 and Fig. 2, the number of plant taxa used by the indeginous community of Afyonkarahisar and the surrounding area is 130 that belong to 93 genera and 39 families, and a total of 178 ethnobotanical uses (remedies) were recorded. Medicinal use occupies the first place with 84 types of use. The others are food with 68, fodder with 16, handicrafts, painting and effects with three types of use each, and other (evil eye) with one. According to results, the percentage of species in families are Asteraceae (14 %), Lamiaceae (10 %), Rosaceae (8 %), Caryophyllaceae (5 %), Chenopodiaceae (5 %), Polygonaceae (5 %), Boraginaceae (4 %), Brassicaceae (4 %), Fabaceae (4 %), and 41 % of them are composed of other subgroups. The richest subgroup rate in terms of frequency of ethnobotanical uses is 15 % Asteraceae, followed by 10 % Lamiaceae, 9 % Rosaceae, 4 % Brassicaceae, 4 % Caryophyllaceae, 4 % Chenopodiaceae, 4 % Fabaceae, 3 % Boraginaceae, and 42 % other subgroups. The richest genus in terms of ethnobotanically significant is *Rumex* L. with 5 taxa, followed by *Quercus* L. with 4 taxa. Seven other 7 genera share thirt place with three taxa each. When we compare the studies of other reseachers [5, 7, 30, 35, 38, 41], the families of Asteraceae, Lamiaceae, Rosaceae are the most common families. But in the study of Doğan [11] the usage order of the families was a bit different than our findings. He reported that the highest number of taxa is similarly Asteraceae, but others were as Boraginaceae, Apiaceae, Lamiaceae, Caryophyllaceae and Geraniaceae. *Rumex* and *Erodium* are the most represented genera.

When the 130 taxa’s usage types are analyzed, it can be seen that the most frequently used parts were leaves (68), flowering branches and flowers (44), fruits (22) and stem (18) (Fig. 2). The usage frequencies of plant parts are observed to be different from local to local [5, 7, 30, 35, 38, 41].

Medicinal use occupies the first place among 178 types of use with 84 remedies. The province of İzmir, Denizli, Ankara, Bilecik, Balıkesir, Muğla are close to our study area. The results of analysis showed that the percentage of the uses shows some similarities. The medicinal plants (47.2 %) are the most cited in Afyonkarahisar. This is almost in agreement with former studies by Ertuğ et al. [7] in Buldan (Denizli) with 42 %, Ertuğ [41] in Muğla with 43 %, Şimşek et al. [5] with 60 % in Ankara, Ugulu et al. [35] with 67 % in Izmir and Güler et al. [30] with 58 % in Bozüyük (Bilecik). These results revealed that local people prefer widely to use the plants for medicinal purposes. The reasons for using the plants widely could be economic, because reaching them easily in folk bazaars and actars with a small amount of money. On the other hand, cultural aspects also play an importan role to use the plants for medicinal purposes.

**Fig. 3:**
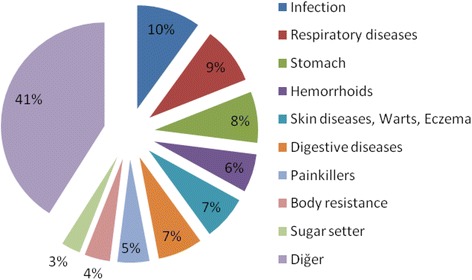
The use aim and usage percentages of the plants in the study area

The rate of food, fodder, others (household goods, dyes, handicrafts and religious) are 38.2, 9 and 5.6 %, respectively. The rates are similar with the studies of Ertuğ [41] in Muğla (38, 15, 5 %) and Şimşek et al. [5] in Ankara (36 %, others (4 %)). According to the data obtained from field work field, plants used by people for infection (10 %), respiration (9 %), stomachache (8 %), skin diseases, wart, eczema (7 %), digestion (7 %), hemorrhoids (6 %), painkiller (5 %), body resistance (4 %), blood sugar regulator (3 %), and other diseases (41 %) (Fig. 3, Table 1). Polat & Satıl [38] reported that various diseases are gastro-intestinal disorders, respiratory and throat diseases, diabetes, kidney ailments, healing cut and wounds, hemorrhoids, anorexia and hypertension stabilizer in Edremit Gulf (Balıkesir). This shows us that the priority of people in using medicinal plants in different localities is different to treat ailments.

We have seen that the culture and ethnobotanical informations that people have gained with centuries of traditional methods are disappearing. Especially today, we can say that increasing of purchasing power and the wealth level has led to a decrease in the use of plants, with more people buying convenience foods, to use cultivated plants, and supplying their medication needs by buying pharmaceuticals from a pharmacy. We determined that in areas where purchasing power is low, people are more prone to ethnobotanical culture.

Particularly, the facts that there are no pharmacies in villages and towns, economic power is low, increased contact with plants, and success in solving some medicinal problems with culture they gained over generations promoting ethnobotanical culture. In comparison to some studies conducted in near areas (in Anatolia), there are some differences in local naming, purpose of use, and how to use plants.

While *Agrostemma githago* “Sakızlık otu” is used in medicinal purposes especially in digestive and alvine conditions in the study region. It is used as ornament along with medicinal purposes [23]. We saw that the plant *Amaranthus retroflexus* was used both as food and for medicinal purposes, especially to treat conditions such as influenza or cold [23]. It was also observed that the plant is called different names such as “Paşa pancarı” and “Kızılbacak” in different localities. *Bellis perennis*, which is called as “Çayır papatyası”, is used for medicinal purposes to treat respiratory diseases. It is also used for cold and flu, stomach-ache, strengthen hair [19]. It is noted that *Capsella bursa-pastoris* “Çoban çantası” is consumed as food by informants, it is also used as food and fodder [1], as food, medicine, fodder and other [23] and as food and medicine [50]. People eat it in meal, roast, soup, or salads [11], cooked as meal with rice and eaten with garlic-yogurt [22]. We note that the purpose of use as food is common in the compared studies.

*Cerinthe minor* subsp. *auriculata* is given to the animals as fodder; people are also reported to use it as food in times of famine [1]. *Chelidonium majus*, called “Kırlangıç otu*”,* is used especially as food and medicine by locals, and it is reported that it benefits conditions related to liver and hemorrhoids. Previous study showed that *Chelidonium majus* is used to treat wart [23]. In some regions, its medicinal uses and purposes differ.

It was reported that *Dianthus zonatus* was used to treat wart by the studies [1, 23, 50] as we found the same purpose. On the other hand, *Ficus carica* L. is used to treat wart in Bozüyük (Bilecik–Turkey) [30]. *Dracunculus vulgaris* is called “Deli otu*”* and is used for infections, and the same aim was reported in the previous studies [1, 23, 50]. It is called as “Yılan bırcağı, köpeksiyen” in Edremit (Balıkesir-Turkey) and used for hemorrhoids, carminative (for animal) [38]. *Hypericum perforatum* shows the same usage as painkiller in the study region and this was repoted in the previous studies [1, 23, 50]. It is also used for stomachache by the report of Güler et al. [30]. While *Portulaca oleracea, “*temizlik otu”*,* is greatly consumed as food in Afyonkarahisar, it is used as salad, pickle and jam in Mersin and Adana provinces (Turkey) [18]. *Urtica dioica,* called “Isırgan”, is used to treat cancer and leukemia by informants. In the other studies, it is used for medicinal purposes [1, 23, 50] and for dye [20, 27]. The plant *Vaccaria pyramidata* var. *grandiflora* is used as fodder in the study region.

Sample survey of some plants is conducted according to compared data. We can come to the conclusion that both local names and uasege purposes of the plants are either the same or vary sometimes. People’s frequency of contact with plants, relation status, passing the plant to next generation, means, and environmental conditions may cause this variety. When we compared some of the plants with some studies in Turkey and in the other countries, we found some differences. While *Anchusa azurea* var. *azurea* is used to treat stomachache, vulnerary, and female sterility as reported in other region [51, 52], we found that it is used as food in the study region. *Capsella bursa-pastoris* is used as an astringent; in burn wound care, for constipation and intestinal spasm, as a diuretic, a hemostatic, and for intestines, kidney swelling, rheumatism, and urinary disorders [23, 53–55], we report that it is used as food. *Peganum harmala* is used for the eliminating the evil eye in our study and used as an analgesic, to treat epilepsy and headache [56], rheumatic pain [44]. *Papaver dubium* is used to treat cold [57], while it is used as food and sedative in our study. *Mentha longifolia* is used to treat halitosis, constipation, common cold, fever, and general weakness and is antispasmodic [58, 59], while it is used to treat Vitamin C deficiency in this study. *Morus alba* is used to treat cancer in our study, while it is used to treat anemia, blood forming, dizziness, hepatitis, incontinence, insomnia, and palpitations in other locals [40, 60]. *Plantago major* is reported to be used by wrapping its leaf around wounded area causing suppuration to flow out. In other studies, it is used to treat, cicatrizer, constipation, hemorrhoids, and wounds [58, 61]. While *Tribulus terrestris* is used to treat athlete’s foot, eczema, kidney and gallstones, hemorrhoids, and warts [38, 62], our study showed that its leaves are consumed by forming wraps. Local people also drink its oil, and it is reported to benefit kidney gravel. The oil of the plant is applied to area affected by hemorrhoids.

*Salix alba* is reported to be used to treat athlete’s foot and vaginal itching [23], we found that it is used to treat pain, stomachache, and respiratory conditions in this study. *Urtica dioica* is used to treat asthma, blood sugar, and intestinal pains, and is used as a diuretic, galactagogue, and post-partum depurative [63, 64] while it is used to treat cancer and leukemia in our study. *Crataegus monogyna* is used to treat respiratory conditions and cold while it is also used to treat arythmia, cardiotonic, diabetes, and is a vasodilator [23].

The majority of the *Origanum vulgare*, *Thymus* spp., *Hypericum perforatum, Achillea millefolium, Rosa canina, Melissa officinalis, Mentha longifolia* etc. species are well known in European folk medicine for their digestive properties, which is also one of the reasons cited for the selection of plants for teas to accompany meals [65, 66]. In the Russian study area, the most used medicinal herbs are *Hypericum perforatum* and *Plantago major*. The Russian respondents considered it important to use medicinal herbs during winter times to prevent flu and common colds [67]. *Amaranthus* spp. [68, 69], *Arum elongatum* and *Lactuca* spp. [70], *Atriplex* sp. [71, 72], *Malva neglecta* [73], *Malva sylvestris* [70, 74], *Morus nigra, Onopordum anatolicum* [70, 75], *Plantago major* [76], *Rumex patientia, Sinapis arvensis* [1, 11, 70–80], *Salvia* spp., *Beta trigyna* [81, 82], *Urtica dioica* [83], the leaves of taxa are used for preparing food (sarma = stuffed food etc.) in the folk cuisines of Turkey and the Balkans. In our study we observed that *Peganum harmala*’ burn incense is believed in to bring about good deed. In the wedding day, the bride and groom are being incensed to get rid off the evil’s harm. It is used in Pakistan for emotional disturbances, painful menstruation, seizures, insanity and itchy skin. Abdominal pain and smoke has insecticidal properties [84].

*Pinus nigra* in Anatolia (spoon making, animal fodder, wetland making), *Cedrus libani* (bowl and spoon making), *Salix alba* (basket weaving), *Juglans regia* (dyes), *Quercus infectoria* (dyes) are used for different purposes [85].

The local names and common families and some species were shared in Anatolia and central Asia (Uzbekistan) [18, 86, 87]. For example, yarpuz/nane for *Mentha* sp., Qoratut/dut for *Morus* sp., itburnu/kuş burnu for *Rosa* sp. (Tablo 1) [87]. In this case, it is a sign that the culture of Anatolia common with central Asia as coming the roots from there.

## Conclusions

This study documented and analyzed traditional ethnobotanical knowledge and 178 different remedies of 130 taxa belonging to 39 families. The results of this study indicated that the local community of the study area used the plants as medicinal (84) and food (68) fodder (16), household goods (3), dyes (3), handicrafts (3) and religious (1). The most common cited usages of plants are still folk medicine and food. Because villagers are generally migrating to big cities and benefiting from the facilities of modern medicine, the heritage of traditional ethnobotanical knowledges is decreasing dramatically. Although this relieve some of the pressures on some plant species, documenting and analizing the indigenous wild plants’ ethnobotanical usages through ethnobotanical studies is still important for the conservation of traditional ethnobotanical knowledge.
